# Sex Differences in Autophagy Contribute to Female Vulnerability in Alzheimer's Disease

**DOI:** 10.3389/fnins.2018.00372

**Published:** 2018-06-22

**Authors:** Erin E. Congdon

**Affiliations:** Neuroscience and Physiology, School of Medicine, New York University, New York City, NY, United States

**Keywords:** Alzheimer, autophagy, sex, sex hormons, tau, amyloid, insulin signaling

## Abstract

Alzheimer's disease (AD) is the most common form of dementia, with over 5. 4 million cases in the US alone (Alzheimer's Association, 2016). Clinically, AD is defined by the presence of plaques composed of Aβ and neurofibrillary pathology composed of the microtubule associated protein tau. Another key feature is the dysregulation of autophagy at key steps in the pathway. In AD, disrupted autophagy contributes to disease progression through the failure to clear pathological protein aggregates, insulin resistance, and its role in the synthesis of Aβ. Like many psychiatric and neurodegenerative diseases, the risk of developing AD, and disease course are dependent on the sex of the patient. One potential mechanism through which these differences occur, is the effects of sex hormones on autophagy. In women, the loss of hormones with menopause presents both a risk factor for developing AD, and an obvious example of where sex differences in AD can stem from. However, because AD pathology can begin decades before menopause, this does not provide the full answer. We propose that sex-based differences in autophagy regulation during the lifespan contribute to the increased risk of AD, and greater severity of pathology seen in women.

## Introduction

Alzheimer's disease (AD) represents a major health crisis that will become of even greater importance as the number of elderly people continues to increase. Currently, over 5 million people have been diagnosed with AD in the US alone (Alzheimer's Association, 2016). The costs and contributions associated with care giving measure in the billions of dollars, further emphasizing the need for a greater understanding its pathogenesis. AD is clinically defined by the presence of two characteristic lesions, the extracellular plaques composed of amyloid-β (Aβ), and the intracellular neurofibrillary pathology made up of the microtubule associated protein tau. However, although both men and women are affected by AD, evidence has emerged that women are at greater risk for both developing the disease, and have more severe pathology (Yoshitake et al., 1995; Fratiglioni et al., 1997; Andersen et al., 1999; Letenneur et al., 1999; Di Carlo et al., 2002; Miech et al., 2002). Indeed, it may be that sex-based differences are the norm for disorders of the CNS, rather than the exception. One possible cause of the differences seen between males and females is the effects of sex, both chromosomal makeup and hormones, on autophagy during the lifetime of the individual. Herein, we propose that the relative vulnerability of women to AD is related to not only the loss of hormones at menopause, but their actions in the preceding decades, as well as inherent differences in expression of proteins in the autophagy pathway.

## Female sex as a risk factor for alzheimer's disease

Sex has emerged as a factor influencing the development and progression of multiple psychiatric and neurodegenerative conditions. Both the incidence, and symptom presentation may differ depending on the sex of the patient. For example, when examining the morphological changes seen in schizophrenia patients, males and females show sex-based variations in brain region volume (Cowell et al., 1996; Narr et al., 2001; Goldstein et al., 2002; Gur et al., 2004). In addition, differences in cognitive and behavioral symptoms have been found (Choi et al., 2009; Esterberg et al., 2010; Fond et al., 2017; Talonen et al., 2017). This effect is also apparent in diseases characterized by abnormal protein deposits. Men show an earlier age of onset in Parkinson's disease, and have lower levels of dopamine transporter in the brain compared to women (for a recent review see Jurado-Coronel et al., 2017). Further, male patients have lower overall motor performance, while women present more frequently with tremors (Jurado-Coronel et al., 2017). Women show more severe motor phenotypes, and faster disease progression, in Huntington's disease (Zielonka et al., 2013). These, and many other findings, suggest that sex-based differences may be the norm for disorders of the CNS.

In the case of AD, the prevalence in the population is higher in women, although there is debate on whether this is due to increased incidence or women's longer life span. Data on whether incidence is higher in women is mixed overall, but several studies suggest that it is in the very elderly (Yoshitake et al., 1995; Fratiglioni et al., 1997; Andersen et al., 1999; Letenneur et al., 1999; Di Carlo et al., 2002; Miech et al., 2002). But, while information on incidence may be less clear, sex-based differences in the effects of risk factors have been found as well. In some cases, these relate to female specific life events, with multiple pregnancies resulting in an increased chance of developing AD (Colucci et al., 2006). Although diabetes raises the likelihood of developing AD for both sexes, incidence in diabetic women is higher than in men (Wang et al., 2012). A well-known AD risk factor, the apolipoprotein E epsilon 4 (APOE-ε4) allele, confers a higher risk of converting from mild cognitive impairment (MCI) to AD on female patients compared to males (Altmann et al., 2014). Additionally, the rate of brain atrophy in patients with probable MCI was faster in women (Hua et al., 2010). Women with a single APOE-ε4 allele also have an earlier age of onset, decreased hippocampal volume, and lower scores on cognitive testing, while male carriers required two copies to show similar detrimental effects (Payami et al., 1994; Fleisher et al., 2005). Decreased cortical volume, more severe hypometabolism, and increased plaque burden compared to male patients with the same genotype have been found as well (Corder et al., 2004; van Helmond et al., 2010; Sampedro et al., 2015). Male carriers of the APOE-ε4 allele have a greater number of cerebral microbleeds compared to women with the same genotype. Interestingly, in transgenic mice this pattern is reversed, which may be due to the higher levels of hypertension in human males, the presence of reactive oxygen species, or the effects of sex hormones on the development of CNS vasculature (Cacciottolo et al., 2016; Finch and Shams, 2016).

Similarly, tau pathology shows marked sex differences as both a risk factor and outcome. Development of neurofibrillary lesions increases the likelihood of displaying cognitive AD symptoms. In men, each additional unit of pathology resulted in a three-fold increased risk, while the risk for women increased 20-fold (Barnes et al., 2005). Further, the pattern of pathology seen at autopsy differs in male and female patients (Schultz et al., 1996; Salehi et al., 1998; Barnes et al., 2005; Koppel et al., 2014). Other tauopathies show sex-based differences as well. In men with frontotemporal dementia, greater glucose hypometabolism was observed (Perneczky et al., 2007), while female patients saw an increased loss of pre-synaptic marker SNAP-25 (Connelly et al., 2011). Women with progressive supranuclear palsy have higher levels of CSF tau than their male counterparts (Wagshal et al., 2014).

Sex-based differences are not restricted to human patients either. Mouse models expressing mutant tau, amyloid precursor protein (APP), or presenilin likewise display sex-based disparities in the severity of pathology. In multiple models, the female sex is associated with higher levels of pathology, and more severe behavioral phenotypes compared to males from the same line (Lewis et al., 2000, 2001; Sturchler-Pierrat and Staufenbiel, 2000; Callahan et al., 2001; Wang et al., 2003; Asuni et al., 2007; Clinton et al., 2007; Yue et al., 2011; Gallagher et al., 2013; Jiao et al., 2016; Buccarello et al., 2017). The deposition of Aβ also affects female mice differently than their male counterparts. Barrier et al. (2010) observed that both male and female mice with amyloid pathology showed increased levels of brain ceremides compared to WT mice. However, the type of ceremide was sex-dependent, with females showing higher levels of 2-hydroxy fatty acid-containing ceramides, and males non-hydroxy fatty acid ceramides (Barrier et al., 2010). Development of tau pathology in response to stress or injury is also exacerbated in female mice (Papasozomenos, 1996; Oikawa et al., 2010; Sotiropoulos et al., 2015).

The use of hormone-based therapy to prevent or treat the cognitive decline seen in AD further highlights the complexity of this issue. Estrogen and testosterone have been shown to have neuroprotective effects in a variety of contexts (reviewed in Engler-Chiurazzi et al., 2017; Merlo et al., 2017), and there is some evidence to suggest that women with AD have lower endogenous estradiol levels compared to non-demented controls (Manly et al., 2000; Yue et al., 2005), but other reports are conflicting (Twist et al., 2000; Cunningham et al., 2001). Lower testosterone levels have been seen in male AD patients compared to controls (Hogervorst et al., 2001; Moffat et al., 2004; Rosario et al., 2011). Both estrogen and testosterone may prevent Aβ and tau pathology, based on experiments in animal models following ovary or testicle removal. Thus, hormone replacement would seem to be an obvious solution. However, in women it appears that treatment must begin shortly after menopause, and continue for some time in order confer beneficial results. Clinical trials in older women (65–79), or women given short term courses of hormones, show no effect (Engler-Chiurazzi et al., 2017; Merlo et al., 2017). In addition, the efficacy of using progesterone in combination with estrogen depends on the route of administration and whether the dosage is constant or cyclical (Engler-Chiurazzi et al., 2017; Merlo et al., 2017).

The greater, and more sudden, loss of hormones that occurs in women during menopause is clearly a major factor in their relative risk compared to men. However, this does not wholly explain the effects seen. We also observe sex differences in animal models. Although mice do experience irregular cycling at 8–9 months of age, and reduced estradiol production, they do not universally undergo a change equivalent to menopause (Brinton, 2012). In addition, AD pathology begins decades before the onset of symptoms, and well before reproductive senescence in women (Braak et al., 2011). Events such as pregnancy that confer increased risk also occur prior to menopause. Thus, differences which occur earlier in life likely lay the foundation for later events. Understanding how an individual's sex contributes to the pathogenesis and progression of AD over their lifetime will provide a better understanding of disease pathogenesis, and may suggest how to best tailor treatment for men and women.

## Sex hormones and receptors in the CNS

The actions of sex hormones are not restricted to reproduction, and affect all tissues including the brain. Testosterone is mainly produced in the Leydig cells in the testes in males, and in the ovaries in females, but is also synthesized in the adrenal glands and brain of both sexes. The corpus luteum and adrenal glands are the main sources of progesterone, with production of estrogens (estradiol, estrone, and estriol) occurring in different tissues. The majority of estradiol is derived from the theca and granulosa cells of the ovaries, while estrone and estriol are mainly synthesized in the liver. However production also occurs in the testes, adipose tissue, and brain (Gruber et al., 2002).

All sex hormones are derived from cholesterol. Steroidogenic cells take up circulating cholesterol via lipoprotein receptors. After entering the cytoplasm, cholesterol is transported to the mitochondrial inner membrane where it is cleaved by cholesterol side-chain cleavage enzyme (P450scc) into pregnenolone. From there, pregnenolone is converted to progesterone by 3β-hydroxysteroid dehydrogenase (3β-HSD), or 17-hydroxypregnolone by 17 α-hydroloase. Either of these two products can be further modified to produce androstenedione and testosterone through a series of reactions involving additional enzymes. Testosterone can be converted to dihydrotestosterone (DHT) by 5α reductase. Estrone and estradiol are produced by aromatization of androstenedione and testosterone, respectively. Which hormones are produced, and by which cells, depends on the presence or absence of enzymes. Further, expression changes during development, with some enzymes present in precursors, but not the mature cells. In adult animals, neurons and glial cells in the olfactory bulb, thalamus, hypothalamus, striatum, hippocampus, cortex, and cerebellum all contain steroidogenic enzymes (Schumacher et al., 2012).

In addition to the enzymes required for steroid hormone production, neurons and glial cells also express hormone receptors. There are three families of progesterone receptors (PR), the first being the classical nuclear progesterone receptors PR-A and PR-B. The two are spliced from the same gene and contain a variable N-terminal, DNA binding domain, variable hinge, and conserved ligand binding domain. These receptors form complexes with heat shock proteins (hsp) 90, 70, and 40 until hormone binding, at which point the receptors dissociate, undergo conformational change, and dimerize (Singh et al., 2013). The second class of PRs comprises a group of seven transmembrane domain G protein-coupled receptors mPRα, mPRβ, mPRγ, mPRδ, mPRε (Petersen et al., 2013). The last group contain a cytochrome b5-heme/steroid binding domain, and include progesterone receptor membrane component 1 (PGRMC1), PGRMC2, neudesin, and neuferrisen (Petersen et al., 2013). The distribution of classical PRs has been well studied, and they can be found in the hippocampus, cortex, hypothalamus, and cerebellum (Singh et al., 2013). The mPRs have a similar spatial distribution, and are found primarily on neurons under normal conditions, but are also expressed on glia following injury (Singh et al., 2013).

The androgen receptor (AR) is similar in structure to the PRs, with a variable N-terminal transcriptional regulation domain, conserved DNA binding domain, and ligand binding domain (Mhaouty-Kodja, 2017). Also like PR, in the absence of hormones, the AR is complexed with chaperones in the cytosol. In adult animals, ARs are found in the hypothalamus, cortex, hippocampus (particularly in the CA1 and CA2/3 regions), amygdala, pre-optic area, and cerebellum (Mhaouty-Kodja, 2017).

Finally, the classical estrogen receptors (ER) ERα and ERβ also share the structure of PRs and the AR. They can be found either in the nucleus, cytoplasm, or associated with the plasma membrane, and form hetero- or homo-dimers upon estrogen binding (Arevalo et al., 2015). A third type, the G protein-coupled estrogen receptor (GPER), is present on the cell surface and has a similar structure to the mPRs. The distribution of ERα and β varies somewhat, with ERα expressed primarily in the pre-optic area, hypothalamus, hippocampus, and to a lesser extent in the cortex, while ERβ is more heavily expressed in the cortex, with additional expression in the hippocampus, olfactory bulb, cerebellum, amygdala, substantia nigra, thalamus, hypothalamus, septum, and ventral tegmental area (Brann et al., 2011).

While the overall distribution of hormone receptors is similar between men and women, sex-based differences in expression are present. In post mortem tissue from the hypothalamus of young adults, similar levels of PRs were found in the superchiasmatic nucleus of men and women (Kruijver and Swaab, 2002). In other brain regions, expression of PRs varies over the course of the estrous cycle, and is stimulated by estrogens in both males and females (Guerra-Araiza et al., 2002; Quadros et al., 2002; Scott et al., 2002; Quadros and Wagner, 2008; Zuloaga et al., 2012). In the case of ARs, men showed much higher levels of nuclear immunoreactivity in the diagonal band of Broca, as well as in the latero- and medial mammillary nuclei than in women. Other hypothalamic regions also showed more staining in men, but the sex differences were less extreme (Fernández-Guasti et al., 2000). Data from ER staining was more mixed. Both overall levels, and intracellular localization of ERs differs between males and females. Young adult women showed stronger ERα nuclear staining in the superchiasmatic, medial mamillary and ventromedial nuclei, while in men nuclear staining was more pronounced in the medial preoptic area, paraventricular nucleus, and lateral hypothalamic area (Kruijver et al., 2002). Male brains also showed higher levels of cytoplasmic ERα (Kruijver et al., 2002). Likewise, differences in ERβ expression and localization were seen. Stronger ERβ nuclear staining was seen in the medial paratenial, paraventricular, and ventromedial nuclei, as well as the medial preoptic area and stria terminalis is men (Kruijver et al., 2003). In the superchiasmatic, supraoptic, infindibular, and medial mamillary nuclei women had higher levels of nuclear ERβ (Kruijver et al., 2003). In nearly all areas where sex differences were seen in cytosolic ERβ levels, staining was more prominent in women. The only exceptions where men had higher levels being the medial preoptic area and dorsal periventricular nucleus (Kruijver et al., 2003).

In animals, sex-based differences have been seen in AR and ER expression on dopaminergic and noradrenergic neurons (Milner et al., 2007; Kritzer and Creutz, 2008; Tao et al., 2012; Rose et al., 2014), in the forebrain (Shah et al., 2004; Milner et al., 2010; Zuloaga et al., 2014), hippocampus (Milner et al., 2010; Tsai et al., 2015), cortex (Milner et al., 2010), and hypothalamus (Lu et al., 1998; Zhang et al., 2002; Shah et al., 2004; Vida et al., 2008; Milner et al., 2010; Kelly et al., 2013; Brock et al., 2015; Jahan et al., 2015; Kyi-Tha-Thu et al., 2015).

Within the brain, sex steroids and their receptors are implicated in a host of processes, including sexual behavior, aggression, mood, cognition, synaptic plasticity, circadian rhythms, and body temperature (Brann et al., 2011; Singh et al., 2013; Arevalo et al., 2015; Mhaouty-Kodja, 2017). They also affect autophagy by themselves, through insulin and neurotrophic signaling, or steroid receptors on mitochondria. In this review, we will discuss how regulation of autophagy differs between the sexes, and further how this may prejudice females toward worse outcomes in AD.

## The autophagy pathway

One way in which sex hormones may influence the development of pathology is through the regulation of autophagy. Under normal conditions, the autophagosome-lysosome system degrades damaged or misfolded proteins and organelles, clearing them from the cell. Within this broad category there are three distinct pathways: macroautophagy, chaperone-mediated autophagy, and microautophagy. Macroautophagy (which will be referred to as autophagy) involves the formation of a double membrane vesicle, with the cargo either surrounded by the elongating structure, or targeted to the autophagosome. This vesicle then fuses with the lysosome. In chaperone mediated autophagy (CMA), proteins containing a KFERQ motif are bound in the cytosol by heat shock cognate and delivered to the lysosome via the lysosome-associated membrane protein (LAMP) 2A. Finally, in microautophagy, the lysosomal membrane itself deforms to engulf the target.

Autophagy progresses through a complex series of interactions (for a recent review see Yu et al., 2017). Briefly, initiation of autophagy involves the formation of several protein complexes: UNC-51-like kinase (Ulk)1/2 complex which also contains Atg13, Atg101 along with several other proteins, a class III lipid kinase complex which includes beclin-1 and phosphatidylinositol 3-phosphate, effector complexes, ubiquitin-like conjugation complexes also containing multiple Atg proteins, and a lipidation complex (Yu et al., 2017). Enlargement of the autophagic vesicle proceeds with contribution from the endoplasmic reticulum and Golgi toward the developing membrane. In addition, lipid droplets also promote autophagosome formation. Fusion with lysosomes requires the further coordination of cellular machinery. First, the vesicles are tethered together, then fusion of the outer autophagosome membrane with the lysosome occurs. Finally, the inner autophagosome membrane is broken down by the lysosomal hydrolases. This requires multiple RAB GTPases, a complex of vacuolar protein sorting-associated proteins, SNAP and SNAREs (Yu et al., 2017).

This process is controlled either in a mammalian target of rapamycin (mTOR) dependent, or independent manner. mTOR forms two protein complexes mTOR complex 1 (mTORC1) and mTOR complex 2 (mTORC2), each with a distinct makeup of proteins (for a review see Laplante and Sabatini, 2012; Wang et al., 2014; Cai et al., 2015). Regulation of mTORC1 occurs through the formation of a dimer of tuberous sclerosis 1 and 2 (TSC1/TSC2), which indirectly inhibits mTORC1 activity. Inhibition of TSC1/TSC2, and thus activation of mTOR, is triggered through a range of signals including growth factors, inflammatory cytokines, and the Wnt pathway. Increased concentrations of amino acids activate mTOR via GTPases in the lysosomal membrane (Sancak et al., 2008; Zoncu et al., 2011) leading to decreased autophagy. DNA damage, hypoxia, and energy deficits may suppress mTORC1 activity through TSC1/TSC2, or directly (Laplante and Sabatini, 2012; Wang et al., 2014). When mTORC1 is active, the downstream results are activation of protein and lipid synthesis, and inhibition of autophagy via phosphorylation of Atg13 preventing it from interacting with Ulk (Laplante and Sabatini, 2012; Wang et al., 2014). Autophagy is also regulated through several mTOR independent pathways. Calcium signaling through the 1,4,5-inositol trisphosphate (IP3) receptor inhibits autophagy, while intracellular calcium, lithium, and trehalose promote it (Zare-Shahabadi et al., 2015). Interestingly, ERα also appears to mediate autophagy in an mTOR independent manner as well (Felzen et al., 2015). In this paper we will review the findings on autophagic dysfunction in AD, and the role of sex on autophagic function. We will focus on several deficits in autophagy found in AD and show how sex-based variations result in the different outcomes seen in men and women come about.

## Sex and autophagy disruption in alzheimer's disease

### Initiation of autophagy

KEY POINTS 1

- Expression of autophagy related proteins is decreased in AD, while mTOR activation is increased.
- Reduced autophagy induction and flux results in failure to clear pathological aggregates.
- Autophagy induction is also affected by sex, with available data suggesting females have lower basal autophagy.
- Lower basal autophagy levels may predispose women to developing greater pathology.

From the earliest time points, data suggests that autophagic flux is lower in females. Expression of autophagy related proteins is lower than in males, and the presence of estrogen and its receptors supress autophagy. AD pathology appears long before either symptoms or menopause, indicating that factors affecting protein clearance early in life likely contribute to the differences in outcomes seen in men and women.

In AD and other neurodegenerative conditions, dysregulation of autophagy results in severe changes compared to healthy cells. This represents a multi-faceted problem involving several stages of the autophagy process. In healthy neurons, autophagy is efficient with distinct patterns of autophagosome formation, and different types of autophagy occurring in different compartments. Typically, autophagosomes in neurons are relatively rare (Nixon et al., 2005), due to rapid fusion with lysosomes and clearance (Boland et al., 2008). In isolated neurons the majority of autophagosome formation occurs in the distal axon, with the vesicles undergoing maturation as they are transported to the soma (Maday et al., 2012; Maday and Holzbaur, 2014). Initiation also takes place to a lesser extent in the soma itself, with very little occurring in the medial axons. Similarly, mitochondrial autophagy is localized to specific areas of the neurons, with damaged organelles trafficked to the soma where they are degraded (Cai et al., 2012).

Both the number and location of autophagosomes are altered in AD. In neurons, the numbers of autophagosomes are vastly increased, with large numbers found in dystrophic neurites as well as in cell bodies (Nixon et al., 2005). This observation demonstrates a clear disruption in autophagic flux, but there is evidence suggesting that induction is impaired as well. Beclin-1 levels decrease over time in the brain during normal aging (Shibata et al., 2006; Lipinski et al., 2010). However, more drastic reductions in the levels of beclin-1 and LC3 were seen in pre-clinical, MCI and AD tissue (Pickford et al., 2008; Tramutola et al., 2015). LC3 is a cytosolic protein which undergoes several cleavage events before being incorporated into the autophagosome membrane (Glick et al., 2010). Further, multiple groups have reported evidence for upregulation of the PI3K-Akt-mTOR pathway in AD brain (reviewed in Tramutola et al., 2017). In support of decreased autophagy induction as a factor in development of AD pathology, data from experiments showing insulin resistance in neurons, as well as the efficacy of mTOR inhibitors, and other autophagy promoters, also suggest a role for decreased autophagy induction. Suppression of autophagy through lowered protein expression or over-activation of mTOR reduces clearance and promotes the deposition of Aβ and tau aggregates, which promotes further dysfunction.

Recent evidence suggests that once Aβ and tau pathology are established, they in turn further inhibit autophagy. In cultured neurons, exposure to exogenous Aβ increases synuclein aggregation via a reduction in autophagy (Lin et al., 2016a). Aβ has been shown to affect autophagy through lowered AMPK activation and impaired insulin sensitivity (Park et al., 2012; Lin et al., 2016a; Seixas da Silva et al., 2017; Chang et al., 2018). Tau fragments can block chaperone-mediated autophagy through incomplete entry into the lysosome. When this occurs, tau remains associated with the lysosomal surface, and can inhibit its function (Wang et al., 2009). Further, tau can bind to tubulin-deacetylase histone deacetylase 6 (HDAC6), a protein involved in both decreasing tubulin acetylation, and autophagosome formation. Increasing tau levels result in both increased acetylated tubulin, and reduced autophagy (Perez et al., 2009). Cells expressing tau show impaired initiation in cells treated with an autophagy promoter (Perez et al., 2009). Thus, the impaired autophagy in AD leads to a buildup of misfolded and aggregated protein. These proteins in turn further depress autophagy initiation, creating a type of positive feedback loop.

These data also suggest a potential factor in the development of more severe pathology in women. As stated above, deposition of pathological proteins begins long before either the appearance of symptoms, or reproductive senescence, suggesting that sex-based differences which occur early in life may have a role to play in the eventual outcome. Available data suggests that overall, females have lower basal levels of autophagy, and that these differences may even begin *in utero*. Several autophagy related genes are located on the X chromosome including: mboa-7 which is involved in incorporating fatty acids into phosphatidylinositol (Lee et al., 2008), histone deacetylase 6 which promotes the fusion of autophagosomes and lysosomes (Mahlknecht et al., 2001; Lee et al., 2010b), dynamin and VMA21 proteins involved in lysosomal acidification (Dowling et al., 2015; Fang et al., 2016), and RAB39B which plays a role in autophagic flux (Cheng et al., 2002; Sellier et al., 2016). However, despite this, females appear to have overall lower levels of autophagy. Human umbilical cord cells from males and females show distinct expression patterns, with male cells having higher levels of beclin-1, and in some studies a higher microtubule associated light chain 3 (LC3) II/I ratio which suggest that autophagy may be more constitutively active in male cells (Addis et al., 2014; Campesi et al., 2016). This same pattern, higher beclin-1 and LC3-II/I in male cells, was seen in cardiac tissue from young rats. In liver cells from these same animals, higher levels of LAMP-1, and a greater colocalization between it and LC3 were seen in males (Campesi et al., 2013). In one study, male rat pups had higher basal LC3II/I than females from the same line (Demarest et al., 2016). A second study found that male rats had significantly higher mRNA levels of LC3 and p62 in muscle and spinal cord tissue between the ages of 1–4 months (Oliván et al., 2014), with protein levels during this time period showing varying sex differences over time.

The presence of sex hormones, and the activation of receptors, also suppress autophagy under basal conditions. Ovariectomized animals, or those lacking ERs show increased basal levels of autophagy in several cell types (Choi et al., 2014; Yang et al., 2014; Camuzard et al., 2016; Yuan et al., 2016; Li et al., 2017; Fu et al., 2018; Tao et al., 2018). In breast cancer cells, reduced expression of ERs, or the use of ER antagonists, promotes autophagy (Cho et al., 2012; Cook et al., 2014). Testosterone deprivation caused by castration can also increase LC3II/I, and inhibit activity of Akt and mTOR (Ibebunjo et al., 2011; Serra et al., 2013; White et al., 2013), while treatment with testosterone can activate Akt and p70S6K (Yin et al., 2009; Ibebunjo et al., 2011). Activation of androgen receptors also activates mTOR via PI3K and ERK (Wu et al., 2010). The upregulation of autophagy seen with hormone and receptor loss is also associated with increased reactive oxygen species, indicating the importance of hormones for the maintenance of mitochondrial health (Park et al., 2016). In addition, nuclear hormone receptors may act directly to regulate transcription of autophagy related genes (Park et al., 2016). Thus, although more research is needed, these data demonstrate how sex differences from the earliest ages may act to predispose women to increased deposition of Aβ and tau. Both chromosomal and hormonal effects influence the rate of basal autophagy, and thus influence the rate of clearance for pathological protein deposits. Lower rates of basal autophagic flux in developing and adult females, and subsequently reduced clearance of early tau and Aβ deposits, may influence both the higher incidence of AD and greater pathology seen in women.

### Insulin resistance

KEY POINTS 2

- Increased insulin resistance is a feature of autophagic dysfunction in AD.
- Both high and low levels of estrogen and/or ER signaling promote insulin resistance.
- Loss of estrogen also impacts Aβ pathology via decreased insulin degrading enzyme levels.

The relationship between estrogen and insulin signaling involves a balance of interactions, and demonstrate how both an excess or loss of sex hormones may cause autophagic impairments seen in AD. In young women, excessive estrogen levels, overactivation of ERs, or abnormal expression of testosterone promote insulin resistance. Following menopause, loss of hormones and decreased receptor expression lead to the same results. In both cases, the result is activation of mTOR and further suppression of autophagy.

Related to the issue of decreased autophagy initiation and activation of mTOR in AD, is the issue of insulin signaling. Multiple groups have found evidence of insulin resistance, and a loss of insulin receptors in AD brain (Moloney et al., 2010; Bedse et al., 2015; Bloom et al., 2017; Tramutola et al., 2017). Indeed, AD itself has been referred to as Type 3 diabetes, and factors which influence insulin signaling may contribute to AD pathology. In addition to effects which may develop over the course of the individual's lifespan, AD pathology itself can induce disfunction in insulin signaling. The presence of Aβ is sufficient to reduce insulin receptors on the cell surface, and inhibit insulin receptor substrate (IRS-1) (Bloom et al., 2017), once again showing how pathology can become self-sustaining once established. Again, like decreased initiation, data regarding the effects of sex on insulin resistance show how factors effecting both young and post-menopausal women contribute to their greater risk for developing AD.

Interestingly, both loss or overactivation of estrogens and ERs can have a detrimental impact on insulin signaling. This shows how the development of pathology may be initiated at earlier ages, with further destabilization of the system occurring at menopause. Generally, sex hormones and receptors are protective against insulin resistance, but this is not always the case. While testosterone is protective in men and can regulate IGF-R1 levels (Ibebunjo et al., 2011), women with higher levels of androgens, such as patients with polycystic ovarian syndrome, are at greater risk for diabetes (Kautzky-Willer et al., 2016). Elevated estrogen levels also represent a risk factor for women in developing insulin resistance (Gupte et al., 2015; Huffman et al., 2017), and progesterone has been implicated in the death of insulin producing cells (Zhou et al., 2013). Further, an ERα variant which increases estrogen signaling has been shown to be a risk factor for developing AD (Boada et al., 2012). Data from the cancer field showed that ER mutations which increase its activity enhance the association of ERs and insulin like growth factor 1 receptor (IGF-1R), and increase IRS-1 phosphorylation (Gelsomino et al., 2016). ERα binding to IGF-1R stimulates the activity of the phosphatidylinositol 3-kinase (PI3K) pathway (Huffman et al., 2017), leading to phosphorylation of Akt, and downstream activation of mTOR. Together these findings show how women could be at greater risk of developing AD. High estrogen or testosterone levels in women can lead to increased activation of ERα, and through it the PI3K pathway. The result being the overactivation of mTOR, and increased inhibitory phosphorylation of IRS-1, leading to insulin resistance. In this way, aberrant sex hormone expression, or receptor activation, may contribute to the development of pathology through their effects on the autophagy pathway over the course of the patient's life.

In addition to the effects of sex hormones during earlier stages, the loss of estrogens and their receptors at menopause also have adverse effects on mTOR via insulin signaling. Both IGF-1 and its receptor have a reciprocal relationship to estrogen, and estrogen and IGF both work to modulate PIK3 signaling (Huffman et al., 2017). Expression of estrogens, and activation of ERs, upregulate the expression and activity of IGF-1R, and IGF binding proteins in neuronal cultures and animals (Huffman et al., 2017). Conversely, inhibition of ERs reduces hippocampal expression of IGF-1R (Huffman et al., 2017). The development of AD pathology itself further contributes to the loss of ERα signaling. ERs interact with tau, and the formation of neurofibrillary pathology can sequester ERα within the tangles (Wang et al., 2016). This may contribute to the vicious cycle in which disruption of autophagy allows the buildup of tau pathology, and tau pathology further inhibits autophagy.

Another component of insulin signaling, insulin degrading enzyme (IDE), is a further connection between this pathway, sex, and AD pathology. Apart from its role in breaking down insulin, IDE also degrades Aβ. Estrogen, progesterone, and testosterone regulate levels of insulin degrading enzyme (IDE) via the PI3K pathway (Udrisar et al., 2005; Zhao et al., 2011; Jayaraman et al., 2012). Ovariectomy resulted in increased Aβ levels in multiple models (Barron and Pike, 2012). It also produced a loss of IDE in the brain, and specifically in the hippocampus, which can be restored with estrogen or progesterone supplementation (Zhao et al., 2011; Jayaraman et al., 2012). This addition of hormones in turn decreased Aβ levels, and this effect was the greatest when progesterone was administered cyclically (Zhao et al., 2011; Jayaraman et al., 2012). These data demonstrate an additional mechanism through which the loss of female hormones contributes to the increased amyloid pathology seen in women (Barron and Pike, 2012). Thus, the loss of estrogen and its receptors can further contribute to the dysregulation of signaling and both too much and too little estrogen can contribute to insulin resistance, and aberrant mTOR activation, starting early in life, and continuing through reproductive senescence.

### mTOR and stress

KEY POINTS 3

- AD features abnormal Ca^2+^ signaling, reactive oxygen species, excitotoxicity and hypoglycemic stress.
- The response to stressors is different in males and females depending on the cell type, and brain region.
- Sex hormones regulate stress induced autophagy through PI3K and AMPK pathways.
- Estrogens also promote autophagic vesicle maturation.

Multiple stressors exist in the brains of AD patients similar to those found during acute brain injury. Data from injury models suggest that female hormones act to stabilize the cells in times of stress, limiting induction of autophagy and promoting its progression. However, when pathology is first initiated, this response may prevent acute toxicity, but hinder clearance. When this process is further destabilized through loss of hormones and receptors, neurons in female brains may be more vulnerable to insult. Decreased estrogen may also result in impaired lysosomal maturation.

AD is a complex pathology with multiple sources of stress contributing to neuronal dysfunction. Aberrant calcium signaling, excitotoxicity, oxidative, and hypoglycemic stressors are all present in the brain of AD patients. Amyloid pathology in particular creates a positive feedback cycle in which the presence of pathology induces calcium influx, and abnormal calcium signaling promotes pathology (Demuro et al., 2010). How the nervous system responds to stress presents yet another potential source of sex-based differences. Overall, data from multiple models suggests that in females both chromosomal makeup and hormones act to suppress stress-induced autophagy and promote vesicle clearance.

Even in the absence of hormones, sex differences are apparent in neurons under stress (for a summary of results regarding chromosome and hormone effects on autophagy see Table 1). XX neurons show no change in autophagy induction following starvation, but rather have more abundant cytosolic lipid droplets. XY neurons in contrast show increased autophagosome formation, as well as higher levels of cell death (Du et al., 2009). Sex-based changes in autophagy following exposure to stressors in the CNS vary between studies. In some models of ischemia, female mice show greater LC3II/I ratios in neuronal and cardiac tissue (Chen et al., 2013; Weis et al., 2014; Demarest et al., 2016), and reduced cell death (Demarest et al., 2016). Females also showed increased mitophagy following injury, while males experienced increased levels of ubiquitinated mitochondrial proteins (Demarest et al., 2016). However, these differences vary depending on brain region and model. Weis et al. (2014) found that neurons in the cerebral cortex, but not hippocampus, of female mice also had higher LC3II than males (Weis et al., 2014). Striatal neurons in males had increased LC3II/I in a brain hemorrhage model (Chen et al., 2012). In addition, knocking down autophagy through inhibition of Atg7 expression was protective for Purkinje cells following ischemia, and this effect was greater in females (Au et al., 2015). Because females have lower basal levels, induction may be more apparent than in males. The variance seen between studies may be due to differences in hormone receptor expression during development and in different areas.

**Table 1:** Chromosome and hormone effects on autophagy.

**XX CHROMOSOMES**		
Basal autophagy	↓ LC3 and beclin	Addis et al., 2014; Oliván et al., 2014; Campesi et al., 2016; Demarest et al., 2016
Stress response	No autophagy induction	Du et al., 2009
**XY CHROMOSOMES**		
Basal autophagy	↑ LC3 and beclin	Addis et al., 2014; Oliván et al., 2014; Campesi et al., 2016; Demarest et al., 2016
Stress response	Increased autophagy	Du et al., 2009
**ESTROGEN**		
Basal autophagy	↑Akt, PI3K, mTOR activity	Cherian and Briski, 2012; Choi et al., 2014; Martínez de Morentin et al., 2014; Yang et al., 2014; Tamrakar et al., 2015; Camuzard et al., 2016; Yuan et al., 2016; Li et al., 2017; Fu et al., 2018; Tao et al., 2018
	↓ AMPK activity	
	↓Autophagy protein expression	
Insulin signaling	↑ IGF-1R activation	Udrisar et al., 2005; Ibebunjo et al., 2011; Zhao et al., 2011; Jayaraman et al., 2012; Li et al., 2015; Huffman et al., 2017
	↑ IGF-1R expression	
	↑ PI3, mTOR activation	
	↑IDE	
Stress response	↑ PI3 kinase activity	Choi et al., 2004; Jover-Mengual et al., 2007; Yang et al., 2010; Chen et al., 2012; Liu et al., 2012; Hsieh et al., 2015; Lin et al., 2016b; Zhang et al., 2017b
	↑Akt, ERK, TrkB activation	
	↓Bcl-2, AMPK activity	
Lysosomal function	↑Maturation	Barron and Pike, 2012; Li et al., 2015, 2017
	↑APPα production	
**PROGESTERONE**		
Basal autophagy	↓Autophagy protein expression	Choi et al., 2014
Insulin signaling	↑ PI3K, mTOR activation	Zhou et al., 2013; Huffman et al., 2017; Jayaraman et al., 2012
	↑IDE	
Stress response	↑ PI3 kinase activity	Singh et al., 2013; Li et al., 2014; Atif et al., 2016
	↑Akt, ERK, TrkB activation	
	↓Bcl-2	
**TESTOSTERONE**		
Basal autophagy	↑Akt, PI3K, ERK mTOR activation	Yin et al., 2009; Wu et al., 2010; Ibebunjo et al., 2011; Serra et al., 2013; White et al., 2013
Insulin signaling	↑IGF-1R expression ↑PI3K, mTOR activation ↑IDE	Udrisar et al., 2005; Ibebunjo et al., 2011; Huffman et al., 2017
Stress response	↑PI3 kinase activity	Huang et al., 2010; Fu et al., 2017
Lysosomal function	↑APPα production	Barron and Pike, 2012

In times of neuronal stress, sex hormones limit increased autophagy by several different mechanisms, the first being the activation of the PI3 kinase pathway. Both estrogen and progesterone prevented autophagy induction in multiple injury models, and activated Akt, ERK, TrkB, and Bcl-2 (Choi et al., 2004; Jover-Mengual et al., 2007; Yang et al., 2010; Chen et al., 2012; Liu et al., 2012; Singh et al., 2013; Li et al., 2014; Hsieh et al., 2015; Atif et al., 2016; Lin et al., 2016b; Zhang et al., 2017b). Expression of ERβ (Hsieh et al., 2015) and GPER (Wang et al., 2017) also reduced autophagy and apoptosis via PI3K pathway activation. Less information is available on the effects of testosterone, but there is data to suggest that it also modulates the PI3K response following injury (Huang et al., 2010; Fu et al., 2017). In addition, estradiol can regulate autophagy through interaction with adenosine monophosphate-activated protein kinase (AMPK). Both male and ovariectomized female mice showed activation of neuronal AMPK in response to hypoglycemia, but this could be prevented through application of estradiol (Cherian and Briski, 2011, 2012; Tamrakar et al., 2015). Ovariectomy alone is sufficient to induce aberrant AMPK activity (Martínez de Morentin et al., 2014). Pharmacological inhibition of AMPK also rescues the phenotype in ovariectomized rats (Tsai et al., 2010). These data indicate that in intact animals, estrogen functions to suppress the activation of AMPK, thus reducing autophagy induction. Finally, Data from Li et al. (2015) suggests that further beneficial effects of estrogens and ER expression may occur through promotion of autophagosome maturation. Upregulation of cell surface ERα, which has been shown to occur in response to injury, or exposure to estrogen, decreased 1-methyl-4-phenylpyridinium (MPP+) induced elevated LC3II, and promoted the maturation of autophagosomes to autolysosomes via transient activation of ERK (Li et al., 2015).

Together, all of these disparate findings add up to another mechanism through which selective female vulnerability occurs. In pre-menopausal women, this suppression of autophagy induction prevents acute cell death caused by exposure to toxic Aβ or tau species. However, by limiting autophagy these proteins are not cleared. Following the dramatic decline in the levels of estrogens and their receptors, other mechanisms come into play. Vesicle maturation is impaired, and neurons become more susceptible to the effects of stressors. In addition, losing the effects of estrogen may also promote pathology through increased AMPK phosphorylation of tau, or Aβ production in the stalled immature autophagosomes.

### mTOR inhibition as potential therapy

KEY POINTS 4

- Inhibitors of mTOR have been used to reduce pathology in AD models.
- The effects of mTOR inhibition are different in males and females.
- In AD models, data from females suggest that interactions between sex and pathology influence efficacy.

As stated in other sections, sex hormones and their receptors have multiple points of contact with the mTOR signaling pathway. Inhibitors of mTOR represent a potential therapeutic option for the treatment of AD, but their use is complicated by sex-based differences in their effects. Data from tests in animals highlights the need to use both sexes in experiments and possibly sex-specific interventions.

Because of the observation that mTOR is aberrantly activated in AD, the use of antagonists as potential therapeutic agents has been explored. Reducing the activity of mTOR by pharmaceutical means, such as rapamycin and its analogs, has been widely shown to improve pathology in animal models of AD (Tramutola et al., 2017). Mice treated with rapamycin typically show induction of autophagy, reduced Aβ and tau pathology, and improvement in behavioral phenotypes (Tramutola et al., 2017). In addition to rapamycin, other mTOR inhibitors have been utilized with similar results; increased autophagy induction, reductions in pathology, restoration of synapses, and improved cognition (Deng et al., 2016; Lin et al., 2017; Tramutola et al., 2017; Zhang et al., 2017a). Few studies have directly compared males and females, but those that have suggest that efficacy of mTOR inhibitors are affected by sex and pathology.

Experiments using models of cardiac hypertrophy, rapamycin function depends on the sex of animal. In females, but not males, rapamycin treatment depressed activity of both mTORC1 and 2, and interfered with the cardioprotective effects of estrogen (Gürgen et al., 2013; Kusch et al., 2015). Very old wild type female mice given rapamycin showed increased expression of molecular chaperones, as well as mTOR, Akt and downstream targets phospho-S6 ribosomal protein (S6) and 4EBP1 (Rodriguez et al., 2014). Chronic rapamycin treatment also extends wild type female lifespan more than male (Miller et al., 2014). Studies using females from AD models alone have been mixed. In one, short term treatment with rapamycin increased Aβ levels in female animals (Zhang et al., 2010). However, rapamycin was effective in females which express APOE-ε4, or mixed amyloid and tau pathology (Zhang et al., 2010; Lin et al., 2017). In all cases there are no males included to compare. Using methylene blue, young female tauopathy mice benefited from mTOR inhibition, but this efficacy was lost in aged females when pathology was well-established (Congdon et al., 2012). In contrast, it remained effective in both young and old males.

Further research is necessary to explore this relationship, but shows that in order to properly assess the efficacy of potential treatment, both sexes must be used. In addition, sex, age, and pathological status can affect the efficacy of mTOR pathway modulators. While mTOR inhibitors may be efficacious in younger women patients, very elderly women may require different interventions.

### Lysosomes and APP processing

KEY POINTS 5

- Common AD causing mutations impact endosomal–lysosomal function.
- Failure of endosome–lysosomes impairs protein clearance and promotes Aβ production.
- Estrogen activates ERK, promoting APPα production, and lysosomal maturation.

As in other aspects of the autophagy pathway, the final stages of lysosome maturation and acidification show why women present with greater pathology than men. In this case, loss of estrogens results in increased Aβ production and a deficit in autophagic flux.

Genetic mutations related to familial and sporadic AD have demonstrated effects on the autophagy pathway as well, and show how menopause negatively affects the late stages of autophagic flux. One of the earliest detectable changes in the endosome-autophagosome-lysosome system in AD is the appearance of enlarged rab5 and seven positive endosomes (Cataldo et al., 2000, 2008). This precedes the development of either clinical symptoms, or Aβ and tau pathology (Cataldo et al., 2000). Further, neurons in the CA1 region of MCI and AD patients show upregulation of rab 4, 5, 7, and 24 (Ginsberg et al., 2010). Increased endosomal activity is associated with increased Aβ production and abnormal localization of lysosomal hydrolases (Grbovic et al., 2003; Cataldo et al., 2008). Endosomal dysfunction is dependent on APP and exacerbated by the presence of the APOE-ε4 allele (Cataldo et al., 2000; Jiang et al., 2010). In addition to these effects, expression of the APOE-ε4 allele in cell and animal models promotes disruption of lysosomal membranes (Ji et al., 2006; Persson et al., 2017), along with increased lysosomal activation and accumulation of Aβ and APOE (Belinson et al., 2008).

Mutations in the presenilin-1 or 2 genes are another source of familial AD cases, and the most common. The presenilin protein has a range of functions in the cell related to its role as a γ secretase, including cell adhesion, regulation of transcription, cell death, cell fate, and neurite outgrowth (for a review see Haapasalo and Kovacs, 2011). In the context of AD, its best-known function is the cleavage of APP. However, it also plays a role in autophagy through the acidification of lysosomes, and the activation of lysosomal proteases. When presenilin is mutated or knocked out in humans and animal models, investigators observe increased lysosomal pathology, and a buildup of proteins in neurons (Cataldo et al., 2004; Esselens et al., 2004; Wilson et al., 2004). Multiple deficits in PS1 knock out cells, including reduced processing and activity levels of cathepsins compared to wild type cells were seen (Lee et al., 2010a). Lysosomes in PS1 deficient cells also fail to fully acidify (Lee et al., 2010a; Wolfe et al., 2013). Similar findings regarding cathepsin and lysosome pH are seen in mice expressing PS1 mutations (Avrahami et al., 2013). The cause of this deficiency in acidification is attributed to the V0a1 subunit of the vesicular ATP-ase proton pump. In order to mature, the subunit must be glycosylated in the endoplasmic reticulum, which requires functional PS1 (Lee et al., 2010a).

In addition, impairment of autophagy can contribute to lesion formation directly. In the brain, endosomes are the primary source of Aβ production. Improper trafficking of APP, amyloidgenic cleavage, and improper fusion contribute to the progression of pathology (Peric and Annaert, 2015). However, the lysosomal compartment also contributes to this process. Experimental data shows that in AD models, APP and BACE1, an enzyme responsible for APP processing, colocalize in lysosomes. Therefore, failure to properly acidify, as occurs with AD mutations, may also increase production (Li et al., 2017). Secretion of Aβ is also heavily influenced by autophagy, with autophagy deficient mice showing reduced secretion, and higher intracellular levels of amyloid (Li et al., 2017). Thus, the current data indicates that both induction and autophagic flux are impaired in AD, limiting neurons' ability to effectively clear, and contributing to, the deposition of pathological protein aggregates.

Menopause, and the accompanying reduction in estrogen, also negatively impacts the final stages of the autophagy pathway. Sex hormones have been shown to affect APP processing through several means. Estrogen and testosterone can affect it directly by lowering beta-secretase (BACE) levels (Barron and Pike, 2012). In addition, they can promote the non-amyloidogenic processing of APP through their effects on autophagy. As stated above, the failure of vesicles to mature and properly acidify is related to the production of Aβ? Treatment of cells with either estrogen or testosterone increases the production of APPα through activation of the ERK pathway (Barron and Pike, 2012). The use of aromatase inhibitors suggested that the effects of testosterone on this pathway required the conversion to estrogen. The activation of ERK by estrogens promotes a shift from autophagosomes to autolysosomes, and autophagy maturation (Li et al., 2015, 2016). Again, these data demonstrate how sex affects the autophagy pathway, and provides an explanation for the differences in risk and outcome between men and women. In women, not only is there impaired clearance, but the loss of hormones directly contributes to the accumulation of Aβ pathology.

## Conclusions

By examining the deficits in autophagy in AD, and the ways in which they intersect with sex, we will gain valuable information on how the sex-based differences seen in AD come about. Women are both at greater risk for developing AD, and develop more severe pathology. This springs from multiple factors spanning the course of the individual's life. Cells containing two X chromosomes express lower levels of autophagy related proteins, while both estrogen and progesterone act to suppress basal levels of autophagy. Women born with highly active ERs, or who suffer from common conditions like polycystic ovary syndrome, are more likely to experience insulin resistance, and the consequent overactivation mTOR. In younger women, neurons and glia respond to stressors by promoting PI3K and inhibiting AMPK. Because the deposition of AD pathology begins as early as childhood, all of these findings show how sex-based differences impact relative risk before menopause. Lower levels of basal autophagy may impair the ability of neurons and glia to remove aggregated proteins, and so too the activation of mTOR caused by insulin resistance. While the dampening of autophagy during stress promotes acute cell survival, further reducing autophagy in the presence of Aβ may contribute to long term pathology.

Women again have increased vulnerability following the decline in hormone and receptor levels after menopause. Because estrogen and insulin signaling are intimately connected, the loss of one produces deficits in the other. Just as too much estrogen produces insulin resistance, so to does too little. Reproductive senescence also contributes directly to lesion formation. Removal of female sex hormones, or receptors, promotes the creation of Aβ through the loss of IDE, and impaired lysosomal maturation. Disinhibition of AMPK can lead to higher levels of phosphorylated tau. Increased pathology can then become self-sustaining through the negative effects of Aβ and tau on autophagy. Further, the efficacy of mTOR inhibitors may vary with age.

The findings presented show how the differing sex-based outcomes seen in men and women in the development and progression of AD may occur. Further study is required to fully elucidate the interactions between sex, aging, and pathology on autophagy, and its role in AD. But doing so will provide greater understanding of the pathogenesis of disease, and how to best to intervene in male and female patients.

## Author contributions

The author confirms being the sole contributor of this work and approved it for publication.

### Conflict of interest statement

The author declares that the research was conducted in the absence of any commercial or financial relationships that could be construed as a potential conflict of interest. The reviewer TN and handling Editor declared their shared affiliation.
